# Analgesia effect of premixed nitrous oxide/oxygen during the rehabilitation after total knee arthroplasty: a study protocol for a randomized controlled trial

**DOI:** 10.1186/s13063-019-3472-7

**Published:** 2019-07-04

**Authors:** Ya-Liang Dai, Xiao-Min Chai, Ning Zhu, Kai-Bin Wang, Wen-Qiang Bao, Xue-Sen Zhang, Lu-Lu Gao, Qiang Liu, Dong-Mei Bao, Li-Ting Wang, Yi-Ling Wang, Jun-Jun Zhang, Yu-Xiang Li, Jian-Qiang Yu

**Affiliations:** 1 School of Nursing, Ningxia Medical University, 1160 Sheng Li Street, Yinchuan, 750004 China; 2 Rehabilitation Department, General Hospital of Ningxia Medical University, Yinchuan, 750004 China; 3 Rehabilitation Department, Workers’ Sanatorium, 581 Zheng Yuan Street, Yinchuan, 750004 China; 4 Pain Department, General Hospital of Ningxia Medical University, Yinchuan, 750004 China; 5 Orthopedics, Wuzhong City People’s Hospital, Wuzhong, 751100 China; 6 School of Preclinical Medical Sciences, Ningxia Medical University, 1160 Sheng Li Street, Yinchuan, 750004 China; 7 Orthopedics, General Hospital of Ningxia Medical University, Yinchuan, 750004 China; 8 Department of Pharmacology, Ningxia Medical University, 1160 Sheng Li Street, Yinchuan, 750004 China

**Keywords:** Analgesia, Rehabilitation, Nitrous oxide

## Abstract

**Background:**

The morbidity of knee arthritis is increasing among aged people and total knee arthroplasty has been its mainstream treatment to date. Postoperative rehabilitation is an important part of the procedure. However, the intense pain during the functional exercise involved has always been a challenge for both patients and health care professionals. The aim of this study is to test the analgesic effect of a mixture of nitrous oxide/oxygeb (1:1) inhalation for patients who are doing functional exercise 1 month after total knee arthroplasty.

**Methods/design:**

This double-blind, randomized, placebo-controlled study will be implemented in the Rehabilitation Department in the General Hospital of Ningxia Medical University. Patients aged between 50 and 75 years who underwent a primary unilateral total knee arthroplasty are eligible for inclusion. The key exclusion criteria include: epilepsy, pulmonary embolism, intestinal obstruction, aerothorax. The treatment group (A) will receive a pre-prepared nitrous oxide/oxygen mixture plus conventional treatment (no analgesics), and the control group (B) will receive oxygen plus conventional treatment (no analgesics). Patients, physicians, therapists, and data collectors are all blind to the experiment. Assessments will be taken immediately after functional exercise begins (T0), 5 min (T1) after functional exercise begins, and 5 min after functional exercise has finished (T2). Patients will be randomly allocated between a treatment group (A) and a control group (B) in a ratio of 1:1. Primary outcome, including pain severity in the procedure, will be taken for each group. Secondary outcomes include blood pressure, heart rate, oxygen saturation, side effects, knee joint range of motion, Knee Society Score (KSS), rescue analgesia need, and satisfaction from both therapists and patients.

**Discussion:**

This study will focus on exploring a fast and efficient analgesic for patients who are doing functional exercise after total knee arthroplasty. Our previous studies suggested that the prefixed nitrous oxide/oxygen mixture was an efficacious analgesic for the management of burn-dressing pain and breakthrough cancer pain. The results of this study should provide a more in-depth insight into the effects of this analgesic method. If this treatment proves successful, it could be implemented widely for patients doing functional exercise in the rehabilitation department.

**Trial registration:**

ChiCTR-INR-17012891. Registered on 6 October 2017.

**Electronic supplementary material:**

The online version of this article (10.1186/s13063-019-3472-7) contains supplementary material, which is available to authorized users.

## Background

Knee arthritis is a chronic joint disease that leads to considerable morbidity in terms of pain, difficulties in performing daily life activities, sleep disorder, functional limitations, mental illness, and decreased quality of life [1, 2]. Knee surgery, including total knee arthroplasty (TKA), for the condition has become increasingly common in developed countries, where it has been influenced a large quantity of aged people occupies 75%. Clinically, TKA is a successful procedure, which is frequently used to bring patients pain relief, improved knee function and quality of life [3, 4]. TKA is associated with damaging postoperative pain that may affect patients’ satisfaction and return to normal function. After TKA surgery, the patients suffer from pain, swelling, restricted blood circulation and a sudden impairment of motor function. Pain levels are consistently reported as higher than other surgical procedures, and are important factors that may influence patients’ willingness to fully engage in rehabilitation activities, which eventually impacts on surgical outcomes [5]. A quick return to normal function after TKA is a desirable outcome for patients, their family members and caregivers. Delayed recovery prevents a prompt return to work and normal activity and increases health care costs [6]. Early mobilization after TKA has been reported to reduce the incidence of complications such as pulmonary embolism, deep vein thrombosis, chest infection, and urinary retention [7, 8], and result in a quicker discharge which could potentially decrease the risk of nosocomial infection [8].

Rehabilitation after TKA generally begins immediately after surgery and, therefore, sufficient analgesia is important. An ideal analgesia for post-TKA rehabilitation will permit adequate knee flexion with minimal pain and without motor impairment, leading to successful mobilization [9]. In China, the development of pain management has lagged that of other countries and has mainly focused on traumatic and acute pain; pain in rehabilitation is not adequately recognized and physicians pay more attention to functional exercise and recovery during the process. Pain relief in rehabilitation post TKA remains a clinical challenge. Patients are required to endure the severe postoperative pain. However, pain-related fear of movement can affect a patient’s functional outcomes [5]. It is hoped that this article provides information that can be used immediately to improve a patients’ experience and outcomes.

Orally administered analgesics are often the main treatment in the immediate- to short-term postoperative period. Despite the effectiveness of opioids, they often lead to some undesirable side effects such as vomiting, constipation, and respiratory depression [10]. It is reported that continuous femoral nerve block (CFNB) with local anesthetics could provide superior analgesia and fewer side effects compared with systemic opioids, thus promoting exercises to increase the knee’s range of motion. Nevertheless, a drawback of CFNB is that it can cause a temporary, reversible weakness that accentuates that already caused by TKA and a subsequent increased risk of falling, thus delaying the ability to move [10, 11]. Nitrous oxide, which has been known as an analgesic gas for more than two centuries, is an effective, fast, safe, and noninvasive form of pain relief in the rehabilitation of patients. Different trials have confirmed that the use of nitrous oxide/oxygen can lead to obvious pain relief in different areas of medicine such as labor, colonoscopy, percutaneous liver biopsy, transrectal biopsy of the prostatic gland, migraine, skin laceration repair, venous cannulation, and dentistry [12–18]. The working mechanism of nitrous oxide/oxygen reported by Emmanouil et al. and Carbajal et al. [19, 20] is that it can stimulate neuronal release of endogenous opioid peptides and activate opioid receptors, aminobutyric acid type A (GABA), and noradrenergic pathways. The anxiolytic and sedative effects resemble those of benzodiazepines. Nitrous oxide hardly dissolves in blood and cannot bind to hemoglobin, acts swiftly and is metabolized by the lungs after inhalation ceases [21]. In 1961, Tunstall developed a stable mixture of nitrous oxide and oxygen for pain relief during childbirth. The two gases were mixed in equal proportions and stored in a single cylinder [22]. Soon, the analgesic use of this gas mixture spread over other settings, such as the dental office, prehospital emergencies, etc. In France, the gas mixture was used only in prehospital care and in some labor rooms, and later in procedure-related pain for children [23]. The mixture could play the same role as at least a 15-mg dose of morphine given intramuscularly [16]. It has been observed that all side effects (nausea, vomiting, dizziness) were transient and vanished within 5 min after removing the inhalation device. No serious side effects were noted [23]. However, this new kind of analgesic has not been accepted all over the world. It is mainly used in developed regions including Britain, Australia, and South Africa. In China, it was firstly used in obstetrics, and later in burns surgery [24]. The apparatus is cheap and portable, but most important is that it is easy for patients to control the device. However, its use in TKA postoperative exercise pain control is rarely reported. This study postulates that a nitrous oxide/oxygen mixture can alleviate the pain level to a certain degree, though not absolutely eliminate the pain of rehabilitative exercise.

## Methods/design

### Patient and Public Involvement

Research questions are based on the PICOS (Population, Intervention, Comparison, Outcome, and Study design) approach:

- Population: patients who undergo total knee replacement and are do functional exercise
- Intervention: nitrous oxide/oxygen inhalation
- Comparison: oxygen inhalation
- Outcome: pain severity, adverse events, satisfaction from both patients and therapists, Knee Society Score (KSS), the time for ROM ≥ 90° and rescue analgesia
- Study design: a randomized controlled trial (RCT) (Additional file 1)

Potential participants for the study will be sourced from the General Hospital of Ningxia Medical University, which is the biggest general hospital in Ningxia. Recruitment sources will be focused on patients who undergo TKA and are doing functional exercise. A member of the research team will contact the principals, directors, case managers and other relevant staff for information about the study. Should they wish their organization to cooperate with the study, staff members will be asked to provide written information to individual members whom they deem to be an appropriate candidate for the study. If that member or client is interested in participating, based on the information provided, they will then voluntarily contact the research team. Alternatively, should the potential participant wish to be contacted directly by a member of the research team, they may choose to complete a “consent to contact” form. At this point, it is made clear that patients are only consenting to the research team contacting them to discuss the possibility of participating in the study as opposed to actually consenting to participate in the study itself. Those who agree to contact the research team will be invited to attend a screening interview to see whether they meet the inclusion criteria (see Table 1). Moreover, the eligible patients will be informed that they do not need to conduct and assess the outcomes, and the specialized researchers will finish these. To maximize participant access, the study will also be advertised via posters. At the time of consent, all study participants will be invited to indicate whether they wish to receive a summary of the findings or not. A written lay summary will be produced and sent to participants. For acknowledging the time and value of participation, participants will be offered a modest reimbursement (for any time and inconvenience associated with participation in the study). The two gases will be offered free for the participants and small “thank you” gifts will also be given.

**Table 1:** Key inclusion and exclusion criteria

Inclusion criteria	Exclusion criteria
Patients underwent primary unilateral total knee replacement	Mental disorder
Age between 50 and 75 years	Drug dependence and drug abuse
Informed and willing to participate in the study	Unconscious and have difficulty in expressing pain by a NRS
Without any postoperative complication	Epilepsy, pulmonary embolism, intestinal obstruction, aerothorax
Recent worst pain (during functional exercise) reported to be 4 or higher (on a Numerical Rating Scale (NRS) of 0 to 10)	Disease involving ear, nose, larynx, such as sinuses, otitis media

### Study design

**Fig. 1:**
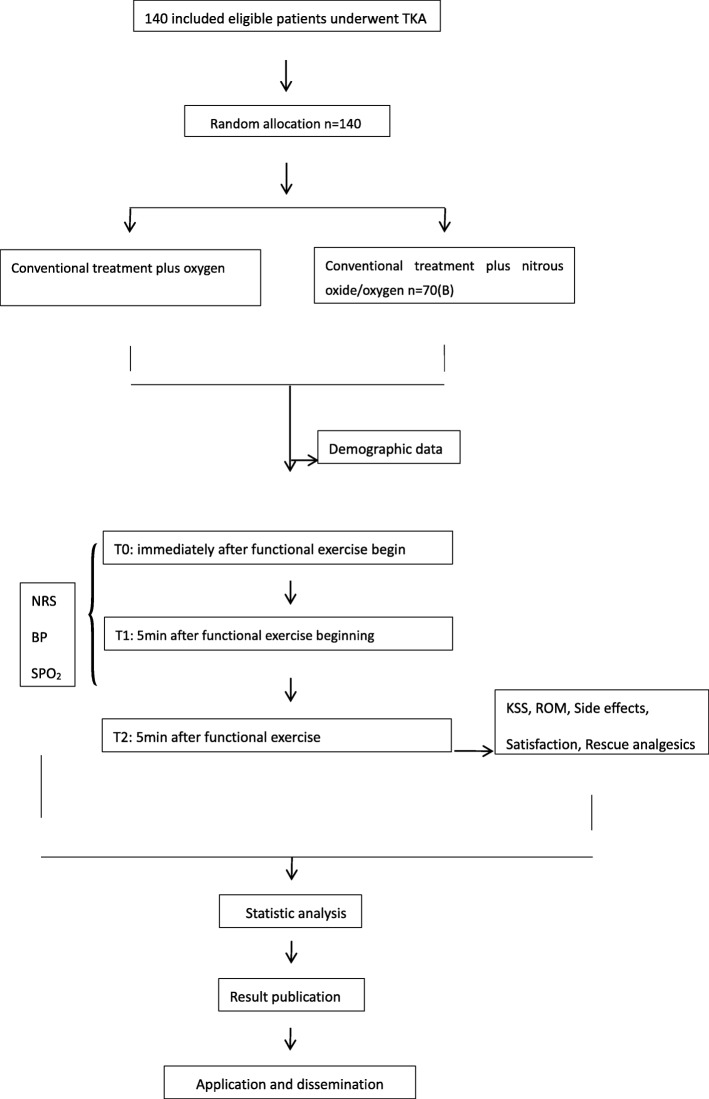
Study design framework. *TKA* total knee arthroplasty, *NRS* Numerical Rating Scale; KSS Knee Society Score, *ROM* range of motion. Physiological indices include blood pressure (BP), oxygen saturation (SPO_2_), heart rate (HR)

This double-blind, randomized, placebo-controlled clinical trial aims to determine the analgesic effect of the premixed nitrous oxide/oxygen used in the rehabilitation department for patients who undergo TKA (1 month after TKA). The study will be conducted in the rehabilitation department of the General Hospital of Ningxia Medical University. Patients in the intervention group will receive conventional analgesic regimens (no analgesic) with the premixed nitrous oxide/oxygen mixture inhalation while patients in the control group will receive conventional analgesic regimens (no analgesic) and oxygen inhalation when doing functional exercise (Fig. 1).

### Study setting and participants

The study will be implemented in the General Hospital of NingXia Medical University, which is a specialized tertiary hospital. A written informed consent form will be collected from all the participants after a thorough explanation of the procedure by the therapists. The researchers state that voluntary participation and all their demographic data and survey answers will be kept confidential. Patients are eligible if they are undergoing primary unilateral TKA (see Table 1). They are ineligible if they are unconscious, or presented with any of the following contraindications to the inhalation of the mixture (hemodynamic instability, vitamin B_12_ deficiency, intracranial hypertension, pneumothorax, intestinal obstruction, epilepsy, pulmonary embolism or facial fractures) [25]. The patients will be randomly allocated to inhale either premixed nitrous oxide and oxygen (intervention group) or oxygen (control group). The medical group and the patients will be all unaware of the mixture inhaled.

### Randomization and blinding

According to the computer-generated random number table in a 1:1 ratio, all the participants will be randomized to the intervention group (conventional treatment plus nitrous oxide/oxygen) or the control group (conventional treatment plus oxygen). The allocation sequence of each patient will be decided by a computer-generated schedule, which will be numbered by a statistician. The randomization schedule will be kept and sealed in an independent research room. Apart from the project manager, who is responsible for gas distribution, no other nurses or data collectors will have access to the data allocation. Prior to, and during, the treatment period, the participants, therapists, and the investigators will all be blind to the allocation. Only the project manager will know the allocation. The two gases will be stored in identical-looking canisters labeled A and B (Additional file 2). Patients given the letter A will be treated using a canister with the appropriate letter containing premixed nitrous oxide/oxygen. Those with letter B will be given oxygen only.

### Procedure

**Fig. 2:**
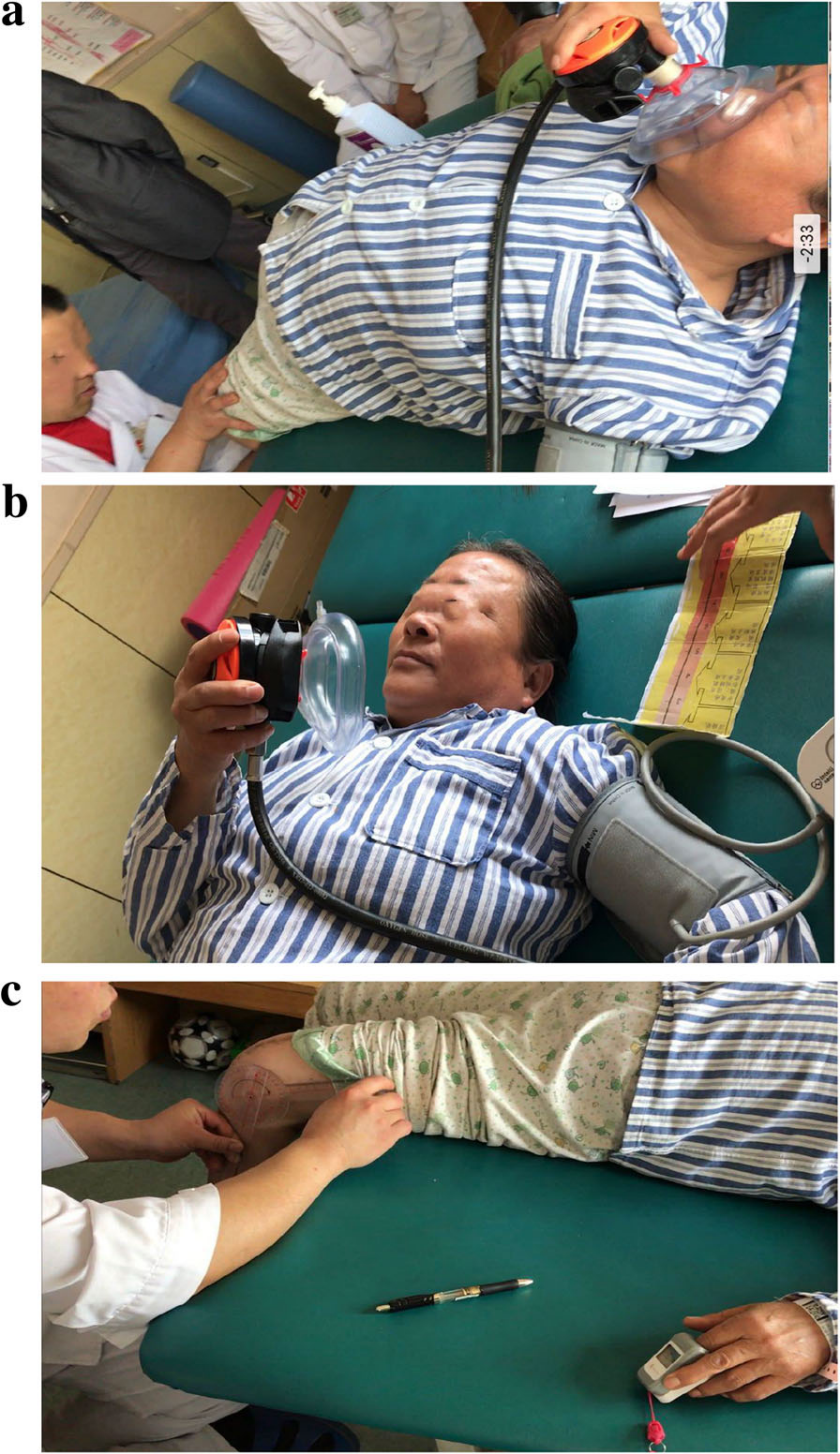
**a** Patient-controlled inhalation of premixed analgesic gas during exercise. **b** Patient’s pain was evaluated with a Numerical Rating Scale (NRS) during exercise, blood pressure was also measured. **c** Active range of motion was measured by goniometer and oxygen saturation of blood was monitored after exercise

All the procedures will be carried out in the rehabilitation department. All the patients will be recruited on the basis of being without any other analgesic before this study. The patients will inhale the premixed nitrous oxide/oxygen or placebo gas through a close-fitting mask for 2 min before exercise and throughout the whole process. They will be continuously monitored with a pulse oximeter during the whole process, and blood pressure will be taken before, during and after each functional exercise session. The inhaled mixture will be administered by the patients themselves (Fig. 2). Neither the therapists nor the patients know which gas mixture will be contained in each gas holder. During the functional exercise, the patients will inhale the gas mixture continuously. The inhalation of the gas mixture will be stopped when the functional exercise ends.

### Measures

The following parameters will be recorded by the performer: age, sex, baseline pain score, blood pressure, heart rate, and oxygen saturation of blood monitored using Infinity Delta XL (Draeger Medial Systems Inc., Danvers, Shanghai, China) [25]. During mixture inhalation, pulse oximetry monitoring will not be necessary because the patients breathe 50% oxygen resulting in hyperoxemia, and the diffusion hypoxia phenomenon does not appear in this type of nitrous oxide use. For safety, we will measure oxygen saturation. Patients will rate their knee pain on the operated side with a numerical rating scale with endpoints of 0 (no pain) and 10 (worst pain tolerable) during flexing [23]. Pain severity, blood pressure, heart rate and oxygen saturation of blood will be monitored immediately after functional exercise begins (T0), 5 min (T1) after the beginning of functional exercise, and 5 min (T2) after functional exercise finishes. Both patients’ and therapists’ satisfaction with pain control under the new analgesia will be investigated by a 5-point satisfaction scale (5, very satisfied; 4, satisfied; 3, uncertain; 2, dissatisfied; 1, very dissatisfied). The KSS [11] will be obtained by questionnaire. According to our previous qualitative study, one of the substantial indicators of discharging from hospital for TKA patients is knee range of motion (ROM) ≥ 90°. Thus, the postoperative knee ROM will be measured by goniometer, and the time to reach ROM ≥ 90° will also be recorded. Moreover, adverse effects, and rescue analgesic need should be recorded and compared.

### Outcome measures

#### Primary outcome

The primary endpoint measures will be pain severity at T0, T1, and T2. The anticipated pain score (Numerical Rating Scale; NRS) during functional exercise will be a reduction from to 3 to 5 in the intervention group. Repeated-measures analysis of variance will be used for data analysis.

#### Secondary outcomes

Secondary outcomes will include physiological parameters: blood pressure, heart rate, oxygen saturation at T0, T1, and T2, adverse effects, and satisfaction investigated by a 5-point satisfaction scale (5, very satisfied; 4, satisfied; 3, uncertain; 2, dissatisfied; 1, very dissatisfied) from both patients and therapists, KSS, and the time for ROM ≥ 90°(ROM will be measured by a goniometer). Rescue analgesia should also be an indicator and recorded.

#### Adverse effects and emergency measures

Respiratory adverse effects, such as desaturation (pulse oximetric saturation≦ 94%), arterial hypotension or bradycardia—as well as hallucination and gastrointestinal adverse effects (nausea, vomiting) or any other discomfort—will be evaluated and recorded during and after the procedure. If any of these happen, patients will be given oxygen inhalation, and the discomfort should vanish within 5 min.

### Sample size determination

Pain relief was defined as a 30% decrease in the pain level in T1-T0  [26]. Based on our preceding study on burn-dressing pain and cancer breakthrough pain [24, 25, 27], we hypothesize that pain relief for 80% of the patients in the nitrous oxide/oxygen intervention group, and 20% of the patients in the placebo control group will be classified as successful. With the threshold for statistical significance set at a *P* value of 0.05 (two-sided alpha), these calculations show that 14 patients are needed (seven per group). We finally decided on a sample size of 140 to meet the Chinese Food and Drug Administration standard for feasibility and the safety of staffs in implementing this analgesic [25, 28].

### Data Safety and Monitoring Board (DSMB)

A DSMB will be established shortly after the project launch and will meet several times during the data collection period. Members will included two pain management specialists; four professional therapists and nursing officers; and a senior academic statistician who served as the Board’s Chair.

### Data analysis

Before the study, all data collectors will be given unified training. The data will be analyzed using SPSS Statistics for Windows, Version 22.0. Chicago, IL, USA. Descriptive statistics will be analyzed by medians (inter-quartile ranges), means (standard deviations), and proportions (exact binomial 95% confidence intervals). The baseline parameters will be compared between the two groups (“control” and “experimental”) using the chi^2^ test or Fisher’s exact test for categorical variables and the Mann-Whitney *U* test for non-parametric data. Statistical significance will be defined as *P* < 0.05.

## Discussion

Knee arthritis is associated with recurrent sharp pain, gait disturbance, joint deformity, and functional degeneration. TKA is the main surgery to solve the problem, but pain along with the need for rehabilitation after TKA has always been a significant clinical challenge for physicians, especially therapists in the rehabilitation department. Endurance has long been strongly represented in the Chinese consciousness. Patients are often told to just get over it, but it's reported that for women patients it is pretty hard [29–31]. Poor pain control results in a lack of timely and sufficient functional exercise which leads to a series of complications, delayed recovery of function, and even the need for a secondary operation. Our preliminary qualitative interview (not published) showed that patients were asked to endure the severe pain but no analgesics were supplied. They bore the pain and tried hard but were unable to do the exercise. In China, especially in the underdeveloped regions, therapists pay much more attention to functional recovery, but inadequate attention is paid to pain control of functional exercise, resulting in untreated or undertreated pain. One important reason is that China was jeopardized by the opium trade and nearly perished in the 1880s. That history is deeply rooted in the heart of every Chinese family and everyone was taught about the Opium Wars in the elementary curriculum and has been taught to avoid from narcotics. Some people even consider the medical use of opioids equivalent to taking drugs. Due to the lack of pain management guidelines, as well as the fear of the adverse effects of analgesia, there was no analgesic action taken for these patients in the rehabilitation department. Patients are given oligoanalgesia and are encouraged to bear the intolerable pain during functional exercise.

The use of adjunct, non-opioid agents is integral for pain control following TKA. The premixed nitrous oxide/oxygen displays its superiority. Its effective use in controlling pain and its rare side effects accelerates its wide use in various areas such as venipuncture, lumbar puncture, bone marrow aspiration, and laceration repairs [21, 23, 32, 33]. To the best of our knowledge, this is the first prospective study on premixed nitrous oxide/oxygen use in rehabilitation after TKA. Abundant literature demonstrates that it is safe for patients themselves to be provided with nitrous oxide analgesia under the supervision of trained therapists [20, 21, 23–25, 27, 34]. Li et al. showed the excellent effect on burn-dressing procedure pain [24]. According to our previous qualitative study and pilot experiment, we hypothesize that this premixed gas will have the same function for patients who undergo TKA. Consequently, we have designed this study with the purpose of exploring the effects of using the premixed nitrous oxide/oxygen for rehabilitation after TKA. If available, it will be applied widely in rehabilitation post TKA and should improve patients’ satisfaction and quality of life.

## Limitations

The limitations of this study must be acknowledged. The study will be conducted in a remote area of China. As a result, our findings may not reflect the national average. Due to the limited resources, the research will be carried out in a hospital only. The results may not represent an ideal research situation and further multi-center studies will be necessary in the future.

## Additional files

Additional file 1: Standard Protocol Items: Recommendations for Interventional Trials (SPIRIT) 2013 Checklist: recommended items to address in a clinical trial protocol and related documents. (DOC 126 kb)

Additional file 2: Equipment. (TIF 379 kb)

## Data Availability

The results of this study will be published in peer-reviewed journals and presented at relevant conferences. Findings will be shared with participating hospitals, policymakers, the academic community, and the general public.
