# Supplementary figures and images for: Analgesia effect of premixed nitrous oxide/oxygen during the rehabilitation after total knee arthroplasty: a study protocol for a randomized controlled trial
Source: Trials. 2019 Jul 4;20:399. doi: 10.1186/s13063-019-3472-7 (PMC6610947; doi:10.1186/s13063-019-3472-7)

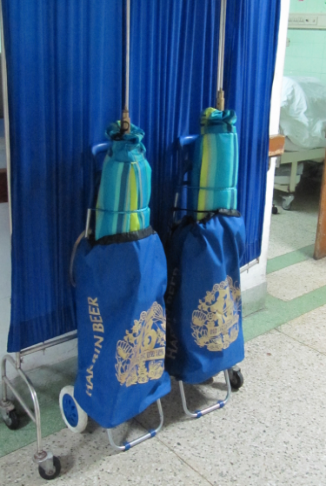

Supplement: Supplementary file 2 — Equipment. (TIF 379 kb) [file 13063_2019_3472_MOESM2_ESM.tif]
